# Assessing Social Inequalities in Older Family Caregivers’ Frailty Conditions, Comorbidity, and Cognitive Functioning: A Cross-sectional Study

**DOI:** 10.1177/23337214231214082

**Published:** 2023-12-21

**Authors:** Roosa-Maria Savela, Tarja Välimäki, Outi Kiljunen, Irma Nykänen, Sohvi Koponen, Anna Liisa Suominen, Ursula Schwab

**Affiliations:** 1 University of Eastern Finland, Kuopio, Finland; 2 Kuopio University Hospital, Kuopio, Finland

**Keywords:** cognitive functioning, comorbidity, frailty conditions, family caregivers, social determinants of health

## Abstract

We aimed to assess the social inequalities in older family caregivers’ frailty conditions, comorbidity, and cognitive functioning. A cross-sectional study was conducted. Study participants were recruited in 2019 in Finland. First, cognitive functioning was assessed with a Mini-Mental State Examination, comorbidity with the modified Functional Comorbidity Index, and frailty conditions were evaluated using the abbreviated Comprehensive Geriatric Assessment. Study participants were also interviewed on socioeconomic factors. The social inequalities in these health outcomes were assessed using the Independent Samples *t*-test, Pearson Chi-square test, and Binary Logistic Regression Analysis. Family caregivers’ (*n* = 125) mean age was 74, and 73% had frailty conditions. Family caregivers’ social inequalities in frailty conditions were linked to their older age and the lowest caregiving cash benefit. Family caregivers’ low educational attainment was also the main factor predicting their minor cognitive impairment. Family caregivers’ social determinants of health should be fully assessed in their health assessment, policies, and programs to ensure healthy aging for both family caregivers and care recipients in the future.

## Introduction

Life expectancy has globally increased by more than 6 years from 2000. Hence, the average life expectancy was around 73 years in 2019, while healthy life expectancy has not followed a similar increasing pattern (World Health Organization, 2020). Moreover, the number of older adults (65 or older) is estimated to rise from around 90 million to nearly 130 million by 2050 across European Union (EU) countries (European Union, 2020). However, the number of adults aged 85 and older is expanding faster than any other age group (European Union, 2020). The increasing number of older adults is challenging for the social and healthcare systems. Therefore, the EU may experience a risk of financially unsustainable healthcare, a long-term care system, and pensions (European Union, 2020).

The current evidence shows that older adults in Europe still experience rather high rates of mental health conditions, chronic illness, disabilities, and frailty in their later lives (European Union, 2020). Frailty may be one of the most complex conditions regarding the aging population. It results from age-related decline in several physiological systems (Clegg et al., 2013), such as decreased physiological capacities (Li et al., 2022). Frailty is linked to decreased functional, psychological, and cognitional dimensions among the older population (Miettinen et al., 2017). Frailty also overlaps with disability and comorbidity and can predict mortality (Clegg et al., 2013). However, it can also be associated with psychosocial factors such as educational level, limited social life, and widowness (Oyon et al., 2021).

Nevertheless, there has been a debate on the definition of frailty. One considers frailty as a biological concept and another as a holistic view, including physical, social, and psychological views of frailty (van Assen et al., 2022). Therefore, there are also different tools to measure the condition. For instance, the Tilburg Frailty Indicator identifies multidimensional frailty, including social, psychological, and physical domains (Gobbens et al., 2010). Other well-known tools are the Fried Frailty Phenotype Scale (Fried, 2016), the Rockwood Clinical Frailty Index (Rockwood et al., 2005), and the Comprehensive Geriatric Assessment (CGA) (Clegg et al., 2013). For example, the Fried Frailty Phenotype Scale measures older adults’ exhaustion, weight loss, slowness, weakness, and low physical activity (Op Het Veld et al., 2015). The Rockwood Clinical Frailty Index is a semiquantitative tool to assess the degree of frailty on a scale of 1 (very fit) to 9 (terminally ill) among older adults (Prendiville et al., 2022). In addition, the Comprehensive Geriatric Assessment (CGA) is a multidisciplinary diagnostic process to evaluate older adults’ psychological, functional, and medical capabilities, a gold standard for assessing frailty (Clegg et al., 2013). However, in this study, we use the abbreviated CGA to evaluate the underlying frailty conditions, including family caregivers’ psychological and functional incapabilities, which may not fully be classified as frailty but can identify the associated conditions. Therefore, we refer to ‘’frailty conditions” throughout the paper. Hence, abbreviated CGA may not be suitable for assessing frailty as a clinical outcome and may not fully diagnose frailty. However, it may be a rather helpful tool to identify those who would benefit from the screening by CGA (Overcash et al., 2005).

Nevertheless, this continuous aging and demographic change across the EU may pressure older family caregivers and their health. For instance, up to 25% of the population in Europe is estimated to provide unpaid care (Zigante, 2018). These caregivers care for their partner, family member, a close friend, or neighbor with various physical and mental health conditions (Schulz et al., 2020). Therefore, family caregivers are essential pillars of society, replacing formal care, notably in Finland (Kalliomaa-Puha & Kangas, 2018). Nevertheless, family caregivers of older adults may also experience adverse health outcomes due to their older age. For instance, a recent study shows that caregivers aged 50 and older may have a greater risk of being frail than non-caregivers (Barbosa et al., 2020). In addition, previous evidence has shown that caregiver burden is associated with an increased risk of frailty among family caregivers (Wennberg et al., 2022). However, although frailty conditions negatively affect individuals’ physical, psychological, social, and cognitive factors, the prevalence of frailty conditions and associated factors among older family caregivers in Finland is less investigated. In addition, many recent studies focusing on frailty have assessed physical frailty (Jiang et al., 2023) or family caregivers caring for individuals with frailty (Ding et al., 2022; Glomsås et al., 2022).

In addition, there is varied evidence regarding cognitive functioning and socioeconomic inequity in general (Cadar et al., 2023) and gaps in the evidence regarding family caregivers’ social inequalities in health (Hepburn & Siegel, 2020; Savela et al., 2022; Young et al., 2020). Therefore, along with frailty conditions, it would also be important to investigate family caregivers’ social inequalities in comorbidity and cognitive functioning. Thus, all these conditions, frailty, comorbidity, and cognitive functioning, interact. For instance, comorbidity may associate with frailty, and increase the risk for cognitive impairment, and disability (Northwood et al., 2018). Then again, cognitive impairment is associated with frailty (Goede et al., 2021). Therefore, frailty should be identified early to prevent or delay its negative outcomes (van Assen et al., 2022).

Addressing the evidence gap on older family caregivers’ social determinants of health may provide important healthcare and policy recommendations to maintain and improve older family caregivers’ health equity, in which the social determinants play a crucial role. Thus, social determinants are the conditions of daily life that may result in health inequalities (Solar & Irwin, 2010). These issues regarding family caregivers’ social determinants of health must be assessed to ensure their care recipients’ good health and well-being. As mentioned, healthy life expectancy has not increased similarly to age (World Health Organization, 2020). Older adults deserve healthy aging, and research should assess the issues which may result in health inequalities among the older population.

Consequently, to better support aging societies, we need evidence of different groups of older adults and their social inequalities in health. Aging societies require a new focus on societal structures and the mechanisms resulting in inequalities (Fritzell et al., 2022). It is time to move from the “one-size-fits-all” approach (Hepburn & Siegel, 2020). Instead, we must address older family caregivers’ social inequalities in frailty conditions, comorbidity, and cognitive functioning to provide future steps to the government’s responses through services and policies to meet future needs. Therefore, this study aims to assess the extent to which social inequalities exist in family caregivers’ frailty conditions, comorbidity, and cognitive functioning with a cross-sectional study approach.

## Methods

This cross-sectional study is based on the LifEstyle, NutriTion, and Oral health in caregivers (LENTO) study’s baseline data, a randomized population-based multidisciplinary intervention study. The methodological details are presented in the study protocol (Nykänen et al., 2021).

### Participants

Study participants were recruited from rural and urban municipalities in Eastern Finland. The recruitment process was active in 2019 from April to early December. The inclusion criteria for participants were those family caregivers with a valid care allowance by the municipality in 2019 and taking care of care recipients aged 65 and older at home. Nevertheless, family caregivers caring for an institutionalized care recipient or those in end-of-life care were excluded. There were no inclusion criteria regarding family caregivers’ age, but they were mainly older adults (98% were 60 and older). In addition, family caregivers mainly cared for their partner (89%), and care recipients were more likely to have memory disorder (63%), which is also the most frequent disease of care recipients under the care allowance (Kalliomaa-Puha, 2018).

Care allowance is a discretionary caring agreement between the municipality and the family caregiver (between the family caregiver and well-being service county since January 2023), including some services, for example, taxable cash benefit for the family caregiver by the municipality. We will refer to this as ‘’caregiving cash benefit”. Family caregivers in the lowest category usually have a care recipient with daily assistance needs and have the lowest cash benefit. In contrast, those in the second category have high-intensive care duties and higher cash benefits than those in the first category but lower than the third category. A similar pattern goes up to the final category.

Municipalities’ workers of social and healthcare services screened the eligible family caregivers using a convenience sampling approach. Hence, all the family caregivers with a valid care allowance were in the municipalities’ registers. They identified 449 eligible family caregivers and provided their contact information for the research team. Then, the research team contacted these family caregivers via mail, and some family caregivers contacted the research team if they wanted to participate. Home visits were then arranged with family caregivers. They received comprehensive oral and written information about the research from the study nurse before the interviews were started. Study participants knew they could withdraw themselves at any time. After the recruitment process, 125 family caregivers participated in the study. The required sample size has been previously assessed (Nykänen et al., 2021).

### Data Collection

**Figure 1.**
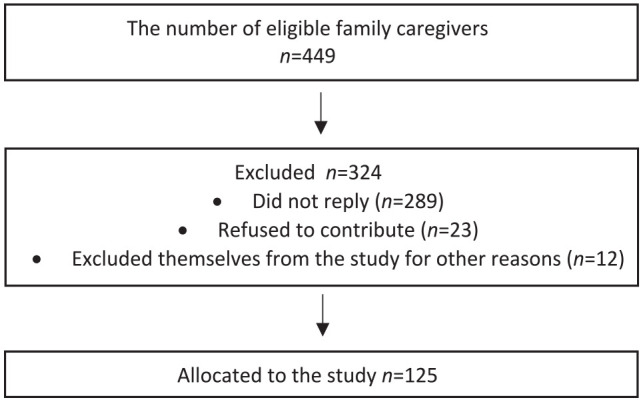
The STROBE (Strengthening the Reporting of Observational Studies in Epidemiology) flow chart of the study sample.

A trained study nurse (the first author) collected data at family caregivers’ households from June 2019 to December 2019. The study participants were able to have the time they needed during the interviews; however, the interviews were approximately 1 hour long. Family caregivers were interviewed on comorbidity, cognitive functioning, and socioeconomic factors and evaluated on frailty conditions. In addition, this study followed the Strengthening the Reporting of Observational Studies in Epidemiology (STROBE) guideline. Please see the flow chart of the study sample in Figure 1.

### Measurements

#### Frailty Conditions

The measurement of frailty conditions is adapted from the previous study conducted in Finland (Miettinen et al., 2017). An abbreviated Comprehensive Geriatric Assessment (aCGA) is based on the full CGA. The aCGA might be the best measurement for community-dwelling older adults in Finland, such as older family caregivers, since this measurement may identify the so-called undetected frailty conditions. On the other hand, CGA is a gold standard for assessing frailty (Clegg et al., 2013), providing validity to use in this study. Prior evidence shows a Cronbach’s *α* of .65 to .92 on CGA and .70 to .94 on aCGA (Overcash et al., 2005). The validated aCGA includes questions from Barthel’s Activities of Daily Living (ADL; scale 0–100) (Collin et al., 1988), Lawton’s Instrumental Activities of Daily Living (IADL; 0–8) (Graf, 2008), Mini-Mental State Examination (MMSE; scale 0–30) (Kurlowicz & Wallace, 1999), and 15-item Geriatric Depression Scale (GDS-15; scale 0–15) (Yasavage & Sheikh, 1986). All these tools are validated and suitable for assessing older adults. For instance, GDS has a 92% sensitivity and 89% specificity regarding diagnostic criteria (Greenberg, 2012).

More specifically, the aCGA assesses frailty conditions based on the questions on the following aspects: four questions on the GDS-15 (“Do you feel pretty worthless?,” “Do you feel that your life is empty?,” “Do you feel happy most of the time?,” and “Do you often feel helplessness?”); three questions on ADL (difficulties in bathing, and the use of toilet and transport); four questions on IADL (unable to prepare food, do shopping, housekeeping, or laundry); and four parts on MMSE (difficulties in calculation, reading, writing, and copying). The frailty conditions based on the aCGA will be scored as follows: a cut-off maximum of ≥1 in functional status (ADL; IADL), ≥2 in depression (GDS-15), and ≤6 in reading, writing, and copying (MMSE). Therefore, family caregivers were assessed as having frailty conditions if they had ≥1 score from one of these domains. Consequently, family caregivers were interviewed on ADL, IADL, MMSE, and GDS-15, and then the aCGA was used to gather the relevant information to assess family caregivers’ frailty conditions. However, we only report outcomes from aCGA, comorbidity, and cognitive functioning among family caregivers in this study.

#### Comorbidity

Study participants’ diseases and comorbidity were assessed using the modified Functional Comorbidity Index (FCI) (Groll et al., 2005; Tiihonen et al., 2018). The modified FCI included 13 diseases, including (1) asthma or chronic obstructive pulmonary disease, (2) cataract, glaucoma, or macular degeneration, (3) coronary heart disease, (4) depressive disorder, (5) diabetes mellitus type I or II, (6) hearing impairment, (7) heart failure, (8) myocardial infarction, (9) osteoporosis, (10) rheumatoid arthritis or other inflammatory connective tissue diseases, (11) stroke, (12) Parkinson’s disease, and (13) dementia. Study participants reported their diagnoses to the study nurse based on the FCI. If the participants consented, the study nurse verified these health conditions from the medical records. We also added obesity to the FCI based on the body mass index (BMI) that the clinical nutritionist of this study measured. Family caregivers’ BMI higher than 30.0 kg/m^2^ indicates obesity. Modified FCI has an inter-rater reliability of 0.55 and an intra-rater reliability of 0.94, while original FCI has 0.61 and 0.90, indicating only a moderate inter-rater reliability in both (Kabboord et al., 2019). Nevertheless, the modified FCI assessed in this prior study used slightly different outcomes. There is little evidence of the validity and reliability of the modified FCI used by Tiihonen et al. However, we may rely on the previous evidence indicating this tool’s validity to assess comorbidity.

#### Cognitive Functioning

As mentioned, family caregivers were assessed on cognitive functioning using the Mini-Mental State Examination (MMSE; scale 0–30) (Monroe & Carter, 2012). Higher scores in MMSE indicated good functioning. The Folstein MMSE is a validated tool to screen cognitive impairment and is often used in geriatric research (Monroe & Carter, 2012). In this study, a cut-point of ≤25 was used for “minor cognitive impairment”. The cut-off value of MMSE is usually set to 23/24. However, prior evidence shows that socioeconomic factors, such as education, could also be acknowledged when considering the cut-point (Arevalo-Rodriguez et al., 2021). In this study, around 48% had 10 years or less education. However, the majority had higher educational levels, and the cut-point was set to ≤25. In addition, family caregivers’ mean MMSE was 26. Previously, the reliability of the MMSE has been measured using, for instance, the 24-hour retest with single and multiple users. The reliability of the MMSE showed a correlation of *r* = .88 by the same testers and *r* = .82 by different testers in 24 hours (Monroe & Carter, 2012). In addition, a Finnish study shows that MMSE has high or good sensitivity and specificity to detect Alzheimer’s disease and mild cognitive impairment, based on the cut-off score of 27 (Nortunen et al., 2018).

#### Socioeconomic Factors

The study nurse interviewed family caregivers on age, sex, municipality (rural/urban), education (in years), income (monthly household net income), and care allowance payment category (category 1 or 2), that is, caregiving cash benefit. Education and income were divided into brackets. For instance, (i) 10 years or less education, (ii) 11 to 15 years, and (iii) 16 years or more, and (i) 2,500 euros per month or less, (ii) 2,501 to 3,500 euros per month, and (iii) 3,501 euros per month or more. These classifications were done based on, for instance, the general education distribution in Finland (Statistics Finland, 2021). Family caregivers were also interviewed on “Have you enough money to meet your needs?” by the World Health Organization Quality-of-Life Brief questionnaire (WHOQOL-BREF) (World Health Organization, 1998). The responses ranged from 1 (no money at all) to 5 (completely enough). The responses were divided into three categories. These were (1) those who reported “no money, a little bit, moderately enough money,” (2) “mostly enough money,” and (3) “completely enough money.”

## Data Analysis

Before data analyses, the normality of continuous variables (i.e., age, education, income, comorbidity, and cognitive functioning) was assessed using the Kolmogorov-Smirnov test. The missing data variables were also assessed. However, only a few missing data variables (non-answered questions) did not require further action. Then, descriptive analysis was used to assess the prevalence of frailty conditions, comorbidity, and minor cognitive impairment. Next, the study participants’ social inequalities were assessed using an Independent Samples *t*-test and Analysis of Variance (ANOVA) or alternatives (Mann-Whitney *U*-test or Kruskal-Wallis test) when continuous variables were involved. Also, we used the Pearson Chi-square test (or Fisher’s exact test) for categorical variables. Binary logistic regression analysis was also used to assess the socioeconomic predictors of frailty conditions, comorbidity, and cognitive functioning. These socioeconomic factors were family caregivers’ age, sex, municipality, education, income, and care allowance category, that is, caregiving cash benefit (category 1 or 2). Multivariable logistic regression analysis, adjusted for these factors, was also used. The adjustments are described in more detail in the results section. After these analyses, the results were summarized using means (M), standard deviations (SDs), counts, percentages, adjusted odds ratios (OR), and confidence intervals (95% CI). We used the *p*-value of equal and <.05 (two-tailed) to indicate significance. Data analyses were conducted using IBM SPSS Statistics (version 27.0).

## Ethical Approval

The LENTO study was conducted in collaboration with the University of Eastern Finland, Kuopio University Hospital, and the municipalities of Kuopio and Vesanto. The research ethics committee of the Northern Savo Hospital District reviewed the ethical issues and gave a favorable opinion to conduct the study. ClinicalTrials.gov NCT04003493.

## Results

### Descriptive Characteristics of Family Caregivers

Table 1 presents the background characteristics of family caregivers. Their mean age was 74, and 73% had frailty conditions. Based on the socioeconomic factors, family caregivers with the lowest caregiving cash benefit had a household net income of 3,023 euros per month versus those with the higher cash benefit of 3,438 euros per month (*U* = 12.437, *p* = .002). This finding indicates income disparities between family caregivers of different caregiving cash benefit categories.

**Table 1.** Socioeconomic Factors and Health Indicators of Family Caregivers (*n =*125).

Characteristics	*n* = 125
Age	
in years (mean, *SD*)	74 (8)
Age brackets	
75 years or younger (*n*, %)	69 (55)
76 years or older (*n*, %)	56 (45)
Sex	
Females (*n*, %)	90 (72)
Males (*n*, %)	35 (28)
Municipality of living	
Urban (*n*, %)	113 (90)
Rural (*n*, %)	12 (10)
Education	
in years (mean, *SD*)	11 (4)
Education	
10 years or less (*n*, %)	60 (48)
11–15 years (*n*, %)	54 (43)
16 years or more (*n*, %)	11 (9)
Household net income (*n* = 124)	
euros per month (mean, *SD*)	3,140 (912)
Household net income (*n* = 124)	
2,500 euros per month or less (*n*, %)	37 (30)
2,501–3,500 euros per month (*n*, %)	54 (44)
3,501 euros per month or more (*n*, %)	33 (26)
Have you enough money to meet your needs?	
Not at all, little or moderately (*n*, %)	38 (30)
Mostly enough (*n*, %)	39 (32)
Completely enough (*n*, %)	48 (38)
Care allowance category (*n* = 123)	
Category 1 (around 400–450 euros gross monthly income) (*n*, %)	86 (70)
Category 2 (around 500–600 euros gross monthly income) (*n*, %)	37 (30)
Health indicators	
Frailty (*n*, %)	91 (73)
Functional comorbidity index, including obesity (mean, *SD*)	2 (2)
Mini-Mental State Examination (MMSE) (mean, *SD*)	26 (3)
MMSE ≤ 25 (*n*, %) “minor cognitive impairment”	38 (30)

### Social Inequalities in Frailty Conditions and Comorbidity

Tables 2 and 3 show the social inequalities in frailty conditions and comorbidity among family caregivers. Family caregivers’ older age (*p* = .03) and lowest caregiving cash benefit (*p* = .01) were linked to frailty conditions. These links were further assessed using multivariable logistic regression analysis. In this analysis, family caregivers’ older age (adjusted OR: 1.0, 95% CI [1.0, 1.1], *p* = .026) and the lowest caregiving cash benefit (adjusted OR: 0.3, 95% CI [0.1, 0.8], *p* = .021) were the only factors predicting frailty conditions. This analysis was adjusted for sex, municipality (rural/urban), education, income, and financial satisfaction (data not shown). In addition, older family caregivers had more comorbidity than younger ones (*p* = .05).

**Table 2.** Social Inequalities in Frailty Conditions Among Family Caregivers.

	Frailty conditions (*n*, %)		*p*-Value
	Yes	No	
Total study population (*n* = 125)	91 (73)	34 (27)	
Age			0.03^a *^
75 years or younger	45 (50)	24 (70)	..
76 years or older	46 (50)	10 (30)	..
Sex			0.12
Females	69 (76)	21 (62)	..
Males	22 (24)	13 (38)	..
Municipality			0.51
Urban	81 (89)	32 (94)	..
Rural	10 (11)	2 (6)	..
Education in years			0.23
10 years or less	47 (52)	13 (38)	..
11–15 years	35 (38)	19 (56)	..
16 years or more	9 (10)	2 (6)	..
Household net income per month (*n* = 124)			0.24
2,500 euros or less	29 (32)	8 (23)	..
2,501–3,500 euros	35 (39)	19 (56)	..
3,501 euros or more	26 (29)	7 (21)	..
Have you enough money to meet your needs?			0.71
Not at all, a little or moderately	29 (32)	9 (27)	..
Mostly enough	29 (32)	10 (29)	..
Completely enough	33 (36)	15 (44)	..
Care allowance category (*n* = 123)			0.01^b **^
Category 1	68 (76)	18 (55)	..
Category 2	22 (24)	15 (45)	..

a Pearson Chi-square.

b Fisher-Freeman-Halton test.

* *p* = .05. **p = .01.

**Table 3.** Social Inequalities in Comorbidity Among Family Caregivers.

	Comorbidity		*p*-Value
	Mean	*SD*	
Total study population (*n* = 125)	2.2	1.6	
Age			0.05^a *^
75 years or younger	1.9	1.3	..
76 years or older	2.6	1.7	..
Sex			0.10
Females	2.1	1.5	..
Males	2.3	1.7	..
Municipality			0.33
Urban	2.2	1.5	..
Rural	2.2	1.9	..
Education in years			0.46
10 years or less	2.2	1.6	..
11–15 years	2.4	1.6	..
16 years or more	1.6	1.2	..
Household net income per month (*n* = 124)			0.61
2,500 euros or less	2.0	1.4	..
2,501–3,500 euros	2.5	1.6	..
3,501 euros or more	2.0	1.5	..
Have you enough money to meet your needs?			0.07
Not at all, a little or moderately	2.4	1.7	..
Mostly enough	2.6	1.6	..
Completely enough	1.8	1.3	..
Care allowance category (*n* = 123)			0.59
Category 1	2.2	1.6	..
Category 2	2.3	1.4	..

a Mann-Whitney *U*-test.

* *p* = .05.

### Social Inequalities in Cognitive Functioning

As seen in Table 4, younger family caregivers (75 years and younger) had significantly better (*p* < .001) cognitive functioning than older ones. Moreover, family caregivers with the highest educational status (16 years or more) had the highest cognitive functioning. The lower the educational level, the lower the score in cognitive functioning (*p* < .001). A similar pattern can be seen among family caregivers with the highest monthly household net income (3,501 euros or more) and those who experienced having completely enough money to meet their needs. These family caregivers’ cognitive functioning was higher than in other financial groups.

**Table 4.** Social Inequalities in Cognitive Functioning Among Family Caregivers.

	MMSE		*p*-Value
	Mean	*SD*	
Total study population (*n* = 125)	26.3	2.8	
Age			<0.001^a ***^
75 years or younger	27.0	2.4	..
76 years or older	25.4	3.0	..
Sex			0.25
Females	26.6	3.0	..
Males	25.8	3.0	..
Municipality			0.52
Urban	26.4	3.0	..
Rural	25.8	2.8	..
Education in years			<0.001^b ***^
10 years or less	25.3	3.0	..
11–15 years	27.1	2.3	..
16 years or more	28.0	1.3	..
Household net income per month (*n* = 124)			0.005^b **^
2,500 euros or less	25.1	3.2	..
2,501–3,500 euros	26.5	2.4	..
3,501 euros or more	27.4	2.4	..
Have you enough money to meet your needs?			0.06
Not at all, a little or moderately	25.9	3.3	..
Mostly enough	25.6	2.9	..
Completely enough	27.2	2.0	..
Care allowance category (*n* = 123)			0.49
Category 1	26.1	2.9	..
Category 2	26.7	2.5	..

MMSE = Mini-Mental State Examination.

a Mann-Whitney *U*-test.

b Kruskal-Wallis test.

** *p* = .01. ***p < .001.

Nevertheless, the multivariable logistic regression model was used to assess the inequalities regarding family caregivers’ cognitive functioning. Therefore, in this analysis, education was the only factor predicting minor cognitive impairment (adjusted OR: 0.72, 95% CI [0.60, 0.87], *p* = .001) when the model was adjusted for age and income. Each additional increase of 1 year in education is associated with a 28% decrease in the odds of minor cognitive impairment (data not shown).

## Discussion

We aimed to assess family caregivers’ social inequalities in frailty conditions, comorbidity, and cognitive functioning. Therefore, the social stratification of family caregivers’ health outcomes should be evaluated. Structural determinants, such as society’s socioeconomic and political context, income, and education, affect intermediary determinants, which may then lead to health inequalities (Solar & Irwin, 2010). Our evidence shows that older family caregivers may have social stratification in their cognitive functioning and frailty conditions. Thus, particularly those family caregivers with the lowest educational status had lower cognitive functioning, and those with the lowest category of taxable cash benefits by the municipality had frailty conditions. This finding may indicate that differences in family caregivers’ educational attainment have led to social stratification among the population, resulting in lower cognitive functioning among those with lower education. For instance, previous evidence shows that education can dominate cognitive functioning among those 50 years and older (Rodriguez et al., 2021), and those with lower years of education may have lower cognitive functioning (Stefler et al., 2021). In addition, in this study, a higher prevalence of frailty conditions among those family caregivers with the lowest taxable cash benefits may be associated with the Finnish political context. The following sub-sections will further assess these issues.

A total of 73% of our study population were identified as having frailty conditions. Although our study results are not comparable to previous studies due to different measurement tools and definitions of frailty, we may still reflect on some previous evidence. For example, a prior Finnish study found a 2% to 24% frailty prevalence based on the index used (FRAIL scale (*n* = 1,152), Rockwood’s Frailty Index (*n* = 1,126), and PRISMA-7 (*n* = 1124)) among community-dwelling older people (Salminen et al., 2020). The 18-year follow-up study also found that frailty was associated with a higher mortality risk (Salminen et al., 2020). Similarly, a systematic review found that prevalence varied from 4% to 59% (participants *n* = 61,500) among community-dwelling older adults (65 years and older) (Collard et al., 2012). The weighted prevalence of physical frailty was nearly 10% and slightly over 13% for the broader definition. The prevalence was higher among women and increased with age (Collard et al., 2012). In addition, another study has shown a greater risk of frailty among caregivers than non-caregivers (Barbosa et al., 2020). The caregiver burden is also associated with an increased risk of frailty (Wennberg et al., 2022). On the other hand, prior evidence from Finland identified that almost 90% of home care clients had frailty conditions based on the aCGA (Miettinen et al., 2017). This result is rather similar to ours. However, the study population is quite different, and further conclusions cannot be made.

Nonetheless, other prior studies have mainly assessed physical frailty (Jiang et al., 2023), family caregivers caring for individuals with frailty (Ding et al., 2022; Glomsås et al., 2022), and family caregivers’ frailty and associated nutritional and physical factors (Kiljunen et al., 2023). Some recent evidence has also assessed the associations between socioeconomic status, frailty, and comorbidity. The evidence showed that socioeconomic status may pose a risk for frailty and comorbidity (Dugravot et al., 2020). On the other hand, a prior study investigated the association between state-level income inequality, individual socioeconomic position, and subjective cognitive decline. The results showed no significant association between income inequality and subjective cognitive decline (Peterson et al., 2019). The researchers indicated that income inequalities alone might not be linked to dementia risk (Peterson et al., 2019). Then again, another recent study stated that socioeconomic inequalities in cognitive functioning exist, although the magnitude varies among cohorts (Stefler et al., 2021). Our study showed income inequalities in cognitive functioning, but in the end, education was the only predictor of minor cognitive impairment. In addition, we found that the lowest category of caregiving cash benefit provided by the municipality predicted family caregivers’ frailty conditions. There was also an income disparity among family caregivers with different caregiving cash benefits. However, there is no previous evidence assessing family caregivers’ cash benefits and the associated health or social inequalities in Finland. Therefore, the results may be difficult to interpret or understand the deeper causes and consequences. Investigating family caregivers’ social health inequalities with a longitudinal study might be necessary to increase knowledge on the topic.

On the other hand, prior evidence from Finland shows that those with high incomes and educational attainment are more likely to buy caring services for their close ones compared to those with low incomes, who usually care by themselves (Kalliomaa-Puha, 2018). This phenomenon can also be considered a socioeconomic issue (Kalliomaa-Puha, 2018). However, the issue is less investigated among family caregivers from different socioeconomic positions, at least in Finland. Then again, a recent European-wide study assessed caregiving arrangements and socioeconomic inequalities. They evaluated the inequalities associated with the Cash-for-Care scheme, which aims to promote informal caregiving by paying so-called “caregiver wages”, and the Care-in-Kind scheme, a publicly funded home care service (Bertogg & Strauss, 2020). The authors found that wealthier households with higher Cash-for-Care payments have a higher probability of outsourcing care (Bertogg & Strauss, 2020). The authors also state that some caregivers consider the Cash-for-Care as “extra cash”. Wealthier caregivers can complement cash allowances with their means, while lower-income households may depend more on the care allowance (Bertogg & Strauss, 2020). This is an interesting phenomenon that should also be investigated more in Finland and assess if the cash benefit provided by the municipality poses inequalities among family caregivers of different socioeconomic positions.

Our findings may have some implications for future family caregiving in Finland. As mentioned earlier, care allowance in Finland is an optional social service, which was switched from municipalities to well-being services counties; therefore, family caregivers’ taxable cash benefits slightly increased (Ministry of Social Affairs and Health Finland, 2022). Nevertheless, more family caregivers may have the lowest caregiving cash benefit since the well-being services counties must standardize the criteria of a care allowance and cash benefits across the country and counties. Then again, the cash benefits still vary across the counties. The lowest cash benefits are around 423 euros, and the highest, around 622 euros per month, within the same caregiving category (i.e., caregivers have similar care tasks) (Carers Finland, 2022). The Informal Care Act states that *“The level of the fee [of caregiving] is determined by the commitment and demandingness of the care.”* However, based on the area of living, family caregivers may receive different amounts of caregiving cash benefits. It is necessary to consider whether the current Informal Care Act creates inequalities among family caregivers in Finland.

Furthermore, it is necessary to consider if some family caregivers would benefit from tax-free cash benefits. Everyone with a care allowance is currently given cash benefits based on county-based guidelines and the caregiving categories, regardless of individual financial needs or circumstances. Therefore, future research should focus more on different cohorts of family caregivers and assess their future social and healthcare needs. Family caregivers’ social determinants of health are also little addressed in Finnish policy discourse or health and policy programs targeted to family caregivers caring for older adults. The current Informal Care Act may also lack both equality and equity. Therefore, increased attention might be needed on the future of family caregiving in Finland.

In addition, as mentioned earlier, the age group of adults aged 85 and older is expanding faster than any other age group (European Union, 2020). This increase will challenge the aging society and family caregivers of older adults and require actions to support sustainable health and well-being among both older adults and their caregivers. Therefore, it could be necessary to assess family caregivers’ frailty conditions, along with other health outcomes by healthcare professionals, at the start of the care allowance. This assessment could also be continuous to ensure family caregivers’ and their older care recipients’ healthy aging. More importantly, family caregivers’ social determinants of health should be fully identified during their health assessment.

Despite this study’s recommendations, several limitations exist. First, due to the study design, we cannot conclude causalities. In addition, we had a generally small sample size. Moreover, 90% of the family caregivers were from the urban municipality. This may have some effects on the results. It may be necessary to have a larger sample size of rural family caregivers and assess their social determinants of health. In this study, with non-significant results, frailty conditions were more common among those living in the rural municipality (83% vs. 72%). Therefore, the results may not show the true association due to the small sample size of rural family caregivers (10% of the study population), which may have posed some errors (Serdar et al., 2021). Nevertheless, this is just conjecture, and the results may be valid. In addition, it would be necessary to assess the study population using the CGA. Thus, aCGA is more likely a helpful tool to identify frailty conditions (Overcash et al., 2005), and CGA could be better for the clinical diagnosis and assessment of family caregivers’ frailty. Moreover, one major limitation is a narrowly assessed social determinants of health among family caregivers. On the other hand, this study also assessed aspects not previously assessed among family caregivers of older adults, including, for example, the Finnish care allowance (social system).

In addition, due to this study design, we cannot evaluate the effect of the study or its practical significance. Moreover, we focused on family caregivers in Finland who were entitled to the care allowance by the municipality. This may have posed a sampling bias. This limitation indicates that our study participants are a specific group of caregivers, and their well-being may differ from that of informal family caregivers. For example, our study sample may have been healthier and had more well-being to participate in the study. Then again, our results may be generalized to older family caregivers with a care allowance in Finland. This study’s strengths also lie in its comprehensively measured concepts by trained study members.

## Conclusion

Older family caregivers may have social inequalities in their cognitive functioning and frailty conditions, indicating a social stratification in their health. Hence, particularly those family caregivers with the lowest educational status had lower cognitive functioning, and those with the lowest taxable cash benefits by the municipality had frailty conditions. Therefore, future solutions for healthy aging require a full assessment of family caregivers’ social determinants of health in healthcare, policies, and programs to improve and ensure their and their care recipients’ well-being.
